# Living at a Geographically Higher Elevation Is Associated with Lower Risk of Metabolic Syndrome: Prospective Analysis of the SUN Cohort

**DOI:** 10.3389/fphys.2016.00658

**Published:** 2017-01-04

**Authors:** Amaya Lopez-Pascual, Maira Bes-Rastrollo, Carmen Sayón-Orea, Aurora Perez-Cornago, Jesús Díaz-Gutiérrez, Juan J. Pons, Miguel A. Martínez-González, Pedro González-Muniesa, J. Alfredo Martínez

**Affiliations:** ^1^Department of Nutrition, Food Science and Physiology, School of Pharmacy and Nutrition, University of Navarra Pamplona, Spain; ^2^Centre for Nutrition Research, School of Pharmacy and Nutrition, University of Navarra Pamplona, Spain; ^3^IDISNA Navarra's Health Research Institute Pamplona, Spain; ^4^CIBERobn Physiopathology of Obesity and Nutrition, Centre of Biomedical Research Network, ISCIII Madrid, Spain; ^5^Department Preventive Medicine and Public Health, University of Navarra Pamplona, Spain; ^6^Cancer Epidemiology Unit, Nuffield Department of Population Health, University of Oxford Oxford, UK; ^7^Department History, Art History, and Geography, University of Navarra Pamplona, Spain

**Keywords:** environmental health, metabolic syndrome, cohort studies, preventive medicine, morbidity

## Abstract

Living in a geographically higher altitude affects oxygen availability. The possible connection between environmental factors and the development of metabolic syndrome (MetS) feature is not fully understood, being the available epidemiological evidence still very limited. The aim of the present study was to evaluate the longitudinal association between altitude and incidence of MetS and each of its components in a prospective Spanish cohort, The Seguimiento Universidad de Navarra (SUN) project. Our study included 6860 highly educated subjects (university graduates) free from any MetS criteria at baseline. The altitude of residence was imputed with the postal code of each individual subject residence according to the data of the Spanish National Cartographic Institute and participants were categorized into tertiles. MetS was defined according to the harmonized definition. Cox proportional hazards models were used to assess the association between the altitude of residence and the risk of MetS during follow-up. After a median follow-up period of 10 years, 462 incident cases of MetS were identified. When adjusting for potential confounders, subjects in the highest category of altitude (>456 m) exhibited a significantly lower risk of developing MetS compared to those in the lowest tertile (<122 m) of altitude of residence [Model 2: Hazard ratio = 0.75 (95% Confidence interval: 0.58–0.97); *p* for trend = 0.029]. Living at geographically higher altitude was associated with a lower risk of developing MetS in the SUN project. Our findings suggest that geographical elevation may be an important factor linked to metabolic diseases.

## Introduction

The metabolic syndrome (MetS) is a cluster of interrelated physiopathological comorbidities identified as a risk factor for cardiovascular disease and type 2 diabetes (Wilson et al., 2005). These factors include obesity (especially central adiposity), hyperglycaemia, dyslipidaemia and hypertension (Grundy et al., 2005). Therefore, this syndrome requires a multiple approach due to its complex and uncertain causes (Grundy et al., 2005; Alberti et al., 2009). The prevalence of MetS is increasing all over the world, closely related to higher obesity and sedentary lifestyle rates (Alberti et al., 2009). Thus the MetS is a public health problem, which could be reduced by means of healthy lifestyle policies and programmes (Lucini et al., 2016).

Despite the fact that some risk factors for developing MetS are widely recognized (e.g., smoking, unhealthy dietary habits, sedentary behavior) (Grundy et al., 2005), insufficient reports about protective factors, such as healthier dietary patterns (Abete et al., 2010), can be found. Concerning modifiable factors, the environment has been suggested to potentially influence the development of metabolic diseases (Friel et al., 2011; Dhurandhar and Keith, 2014; Valdes et al., 2014). Moreover, some studies on subjects living at high altitudes have reported lower incidence rates of conditions linked to MetS such as obesity (Voss et al., 2013; Woolcott et al., 2014; Diaz-Gutierrez et al., 2016), heart disease (Ezzati et al., 2012; Faeh et al., 2016), hypertension (Norboo et al., 2015) or type 2 diabetes (Woolcott et al., 2014).

Previous trials have assessed the role of hypoxia on metabolic and physiologic characteristics (Millet et al., 2016). For instance, body weight was significantly lower in obese subjects exposed to hypobaric hypoxia at geographical altitude (Lippl et al., 2010; Vats et al., 2013). Furthermore, increased basal metabolic rate and leptin levels together with decreased food intake and diastolic blood pressure have been associated with the exposure to higher altitudes (Westerterp-Plantenga et al., 1999; Lippl et al., 2010). The previously mentioned clinical trials suggest that not only normobaric hypoxic exposures, but also hypobaric hypoxia could drive some beneficial effects commonly associated with living at geographical altitude. Some studies have reported beneficial effects of a short-term geographical altitude exposure in subjects with MetS (Schobersberger et al., 2003; Greie et al., 2006; Neumayr et al., 2014; Gutwenger et al., 2015). On the other hand, some investigations have reported non-significant changes in obesity or cardiovascular risk factors in obese individuals treated with sham intermittent hypoxia during exercise (Gonzalez-Muniesa et al., 2015b,c; Gatterer et al., 2015) or without exercise (Querido et al., 2012). Likewise, the effects of exercise recovery in hypobaric hypoxia were analyzed in previous studies, with no statistically significant results (Gatterer et al., 2015). Few studies have observed a reduction in body weight of obese subjects treated with physical exercise under normobaric intermittent hypoxia compared to sham hypoxia group (Netzer et al., 2008; Kong et al., 2014). However, higher oxygen availability has been related with beneficial effects in several clinical disorders with an underlying inflammatory cause (Gonzalez-Muniesa et al., 2015a). However, to our knowledge, no prospective study has analyzed the association between living at different altitudes and the risk of MetS in healthy participants. This analysis hypothesized that living at a geographically higher elevation may protect against metabolic syndrome. Hence, the aim of the present study was to evaluate the longitudinal long-term association between altitude of residence and incidence of MetS in a prospective cohort of Spanish university graduates.

## Methods

### Study population

**Figure 1:**
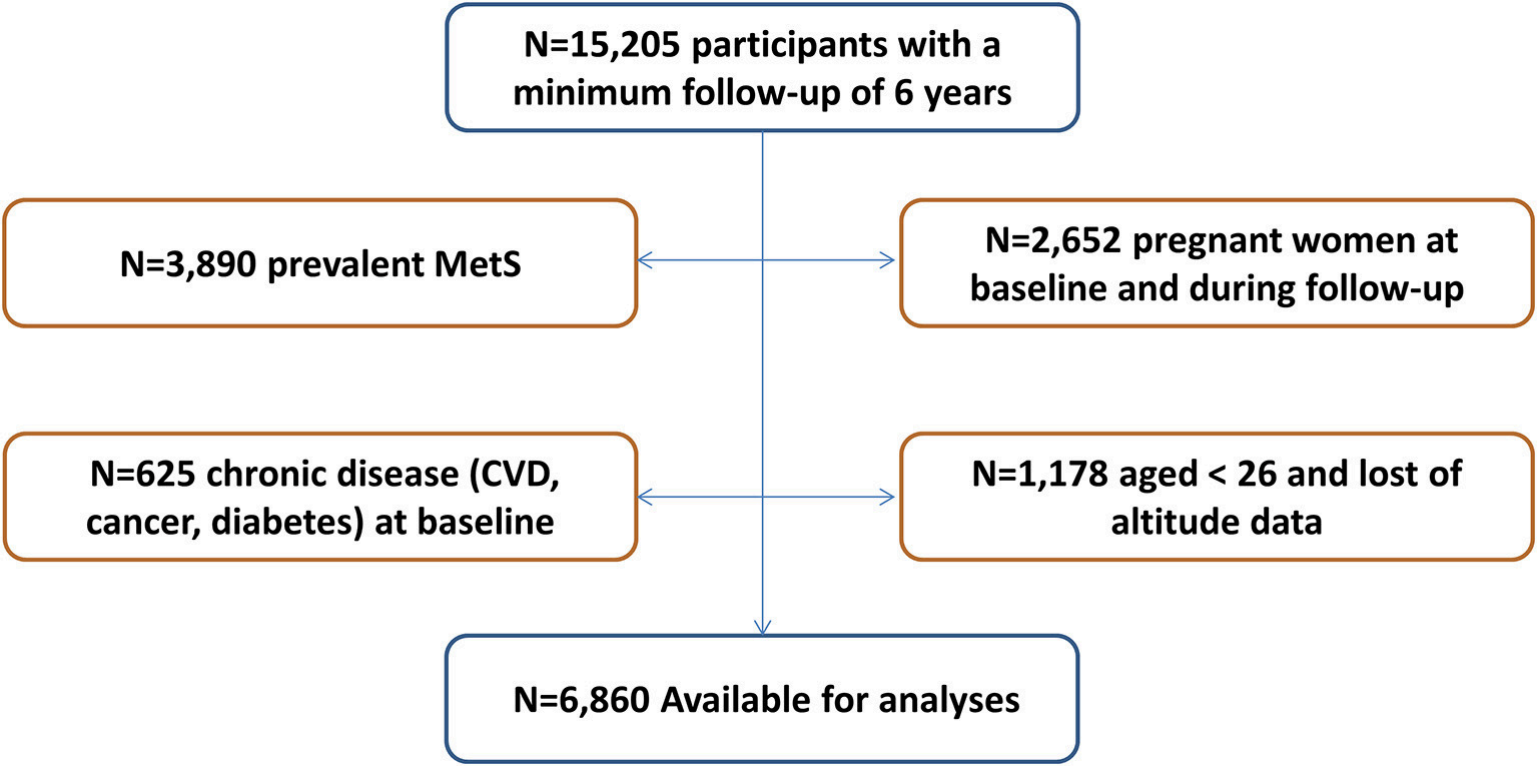
**Flow-chart displaying the participants included in the analyses**. N, number of subjects; MetS, Metabolic syndrome; CVD, cardiovascular disease.

The SUN project (Seguimiento Universidad de Navarra, University of Navarra Follow-up) is a prospective, dynamic, multipurpose cohort study conducted in Spain. The objectives, design and methods of the SUN cohort have been previously described (Martinez-Gonzalez et al., 2002; Martinez-Gonzalez, 2006; Segui-Gomez et al., 2006). Briefly, using biennial mailed questionnaires participants have been continually followed-up. Participants' recruitment started in December, 1999 and it is permanently open. All subjects have obtained a university degree. Up until March, 2012 to have enough time for a 2-year follow-up, the SUN project recruited 21,291 participants. The subjects that ceased to respond the questionnaires of the study (*n* = 1881) were considered lost to follow-up leaving a total of 19,410 (retention rate = 91%). Participants who had not a minimum follow-up of 6 years were excluded from the study (*n* = 4205). To avoid reverse causality bias, 3890 individuals who met at least one MetS criterion at baseline were excluded. Pregnant women at baseline and during the follow-up were excluded (*n* = 2652). In addition, participants suffering from a chronic disease (diabetes, cancer or cardiovascular disease) at baseline were also excluded (*n* = 625). For the present study, a subsample of the cohort was selected for the final analysis with a total of 6860 participants over 25 years old that had answered the postal code in the baseline questionnaire (Figure 1). The study was conducted according to the guidelines laid down in the Declaration of Helsinki and it was approved by the Human Research Ethical Committee of the University of Navarra. Voluntary completion of the first self-administered questionnaire was considered to imply informed consent.

### Assessment of habits and lifestyles

The baseline questionnaire collected subjects' clinical history, daily habits, lifestyle, social characteristics, the time of residence at the current city and self-reported anthropometric information. Leisure-time physical activity was calculated through a baseline 17-item questionnaire. The reproducibility and validity of self-reported physical activity was assessed in a subsample of the cohort (Martinez-Gonzalez et al., 2005). Adherence to the Mediterranean dietary pattern was determined through the score (0–9 points) proposed by Trichopoulou et al. (2003).

### Assessment of altitude

The postal code of each participant's residence and the time they have been living in their city/village was recorded using the baseline questionnaire. We imputed the altitude of each postal code according to Spanish National Cartographic Institute (Ministry of Public Works-Ministerio de Fomento, Spanish Government) using the program GIS ArcGIS v10.3 and categorized participants into tertiles.

### Assessment of metabolic syndrome

MetS was defined according to the International Diabetes Federation and American Heart Association/National Heart, Lung, and Blood Institute harmonized definition (IDF-AHA/NHLBI) (Alberti et al., 2009). According to it, the MetS diagnosis requires the occurrence of at least three of the following five criteria: central adiposity (according to the country-specific definition waist circumference: ≥94 cm in males and ≥80 cm in females), hypertriglyceridemia (≥150 mg/dL or specific medication), low levels of high-density lipoprotein cholesterol (HDL-c: <40 mg/dL for men and <50 mg/dL for women), elevated blood pressure (BP: systolic ≥130 and/or diastolic ≥85 mmHg or antihypertensive drug treatment in a patient with a history of hypertension), and impaired glucose metabolism (fasting glucose ≥100 mg/dL or drug treatment of elevated glucose).

For a comparison between different MetS diagnosis criteria AHA/NHLBI, IDF and Adult Treatment Panel III (ATP III) MetS definitions were used (Alberti et al., 2009). These requirements differ from those in the harmonized definition in the following terms: AHA/NHLBI defines central adiposity when waist circumference ≥102 cm in males and ≥99 cm in females or when diagnosed of type 2 diabetes; IDF specifies the central adiposity as mandatory criterion and at least two of the four criteria (equal to harmonized definition items); ATP III defines central adiposity as waist circumference ≥102 cm in males and ≥88 cm in females.

Self-reported information about each specific MetS criterion was collected in the Q_6 and Q_8 questionnaires (6th and 8th year follow-up respectively). Additionally, a measuring tape was sent to each participant including an explanation of how to measure their own waist.

Diagnosis of MetS itself, the same as each MetS criterion was previously validated in a subsample of the cohort, finding an agreement between self-reported MetS and MetS diagnosis of at least 90% (Barrio-Lopez et al., 2011).

### Statistical analyses

Estimated tertiles according to their baseline altitude of residence were used to classify the participants included in the study: 0–121 meters (m) as the reference level, 122–456 m and ≥457 m. Baseline characteristics according to their altitude of residence were described using relative frequencies, means and standard deviations.

Cox proportional hazards models were used to assess the association between the tertiles of altitude level and the risk of MetS during follow-up. Person-time of follow-up was calculated for each participant, from the date of completion of the baseline questionnaire until the date of completion of the last follow-up questionnaire, or date of death, whichever occurred first. For the cases of incident MetS, the person-time follow-up was set as the date of the follow-up questionnaire where they met the criteria for MetS diagnosis. The Cox model included age as the underlying time scale for all analyses. All models were stratified by years living in the city/village, categories of participants according to their date of entry into the cohort and categories of age (deciles). An initial model adjusted for gender was calculated. Two multivariable-adjusted models were fitted for potential baseline confounders. Model 1 was adjusted for gender, BMI (Kg/m^2^), total energy intake (Kcal/d), adherence to Mediterranean dietary pattern (0 to 9 score), physical activity (METs-h/week), sedentary behavior (h/d), hours sitting (h/d), smoking status (non-smoker, smoker, former smoker), snacking between meals (yes/no) and following special diets (yes/no). Model 2 was additionally adjusted for sleeping time (h/d) and alcohol intake (g/d).

Tests for linear trends across increasing categories of altitude were conducted by assigning the median altitude of residence within each category and treating this variable as continuous. The specific relationship between each of the 5 components of the MetS and the altitude levels was assessed. As sensitivity analyses, the risk of developing MetS living at geographically higher altitude was calculated excluding participants with family history of chronic diseases such as obesity, diabetes, stroke and high blood pressure. Hazard Ratios (HR) and their 95% confidence intervals (CI) were calculated. The proportional-hazards assumption was tested with the Schoenfeld residual method and time dependent covariates.

All *p*-values presented are two-tailed; *p* < 0.05 was considered statistically significant. Analyses were performed using STATA/SE version 12.0 (StataCorp, College Station, TX, USA).

## Results

The main characteristics of participants according to their altitude of residence are shown in Table 1. Higher altitudes on average were associated with younger age, greater proportion of women, lower BMI and less physical activity. No significant differences were found in baseline sedentary behavior, smoking status, alcohol consumption, adherence to the Mediterranean dietary pattern, special diets or sleeping time. Participants in the highest category of altitude had higher energy intake and were more likely to snack between-meals.

**Table 1:** **Baseline characteristics of participants according to their altitude of residence**.

	**Altitude level**			***p*-value^a^**
Range (m)	0–121	122–456	457–2297	
Median (m)	43	330	591	
Mean (m)	48	317	635	
Participants (n)	2308	2374	2178	
Age (y)	41 (9)	40 (9)	40 (9)	0.033
Women (%)	52.9	58.1	56.7	0.001
BMI (Kg/m^2^)	23.4 (2.7)	23.2 (2.7)	23.2 (2.8)	0.012
Physical activity (METs-h/week)	23.2 (22.7)	21.7 (23.3)	21.4 (24.7)	0.019
Sitting time (h/d)	5.2 (2.0)	5.2 (2.1)	5.2 (2.1)	0.372
Smoking status (%)				0.823
Current smokers	19.9	21.2	21.6	
Former smokers	31.2	29.7	30.2	
Total energy intake (Kcal/d)	2467 (819)	2542 (882)	2556 (930)	0.001
Alcohol (g/d)	7.0 (10.5)	6.7 (9.7)	7.0 (9.8)	0.498
Mediterranean dietary pattern^b^	4.3 (1.8)	4.2 (1.8)	4.2 (1.8)	0.242
Between-meals snacking (%)	28.1	31.4	29.2	0.045
Following special diets (%)	6.8	6.0	5.2	0.086
Sleeping time (h/d)	7.3 (0.8)	7.3 (0.8)	7.3 (0.8)	0.758

*The SUN Project 1999–2012. Values are presented as mean (SD) when a percentage symbol (%) is not specified. m, meters; n, number of subjects; y, years; MET, metabolic equivalent task*.

a *p-value for comparison between-groups calculated by one-way ANOVA for continuous variables or the χ2 test of linear trend for categorical variables*.

b *Trichopoulou score (range of scores, 0–9, with higher scores indicating greater adherence)*.

Participants were followed-up for a median time of 10 years. During the follow-up period 462 (6.73%) incident cases of MetS were observed (Table 2A). Subjects in the highest category of altitude (>456 m) exhibited a significantly lower risk of developing MetS during follow-up compared to those in the lowest category (<122 m) of altitude of residence [Model 2: HR = 0.75 (95% CI: 0.58–0.97); *p* for trend = 0.029]. Participants who lived between 123 and 456 m did not show significantly lower risk of developing MetS compared to those in the tertile 1. Moreover a linear trend was observed (*p* = 0.029 in Model 2). In addition to this, a multivariable linear regression showed that for every additional 100 meters of altitude the MetS incidence (each MetS criteria considered qualitatively on yes or no with a maximum of 5 points) is expected to decrease slightly, but significantly (*p*-value = 0.003), in the 8-year follow up.

**Table 2A:** **Associations between altitude and incidence of MetS**.

	**Altitude levels**			***p* for trend**
	**0–121 m**	**122–456 m**	**457–2297 m**	
Participants (n)	2308	2374	2178	
No. of cases per person-year	174/18,285	161/18,977	127/17,413	
Gender	1.00 Ref.	0.93 (0.74–1.17)	0.77 (0.60–0.98)	0.037
Model 1^a^	1.00 Ref.	0.91 (0.72–1.16)	0.76 (0.59–0.98)	0.035
Model 2^b^	1.00 Ref.	0.90 (0.71–1.15)	0.75 (0.58–0.97)	0.029

*The SUN Project 1999–2012. Hazard Ratios and 95% CI of incident MetS (Harmonized IDF-AHA/NHLBI) according to the altitude of residence*.

*m, meters; n, number of subjects*.

a *Model 1 adjusted for: gender, BMI, total energy intake, adherence to Mediterranean dietary pattern, physical activity, sedentary behavior, hours sitting, smoking status, snacking between meals and following special diets*.

b *Model 2 additionally adjusted for sleeping time and alcohol intake*.

The null hypothesis that the relative hazard was constant over time was confirmed, as a non-significant result was found (*p* = 0.19). The time-dependent covariates were tested using this method, which gave a non-significant result indicating a non-zero slope, and confirming that the relative hazard is constant over time.

**Figure 2:**
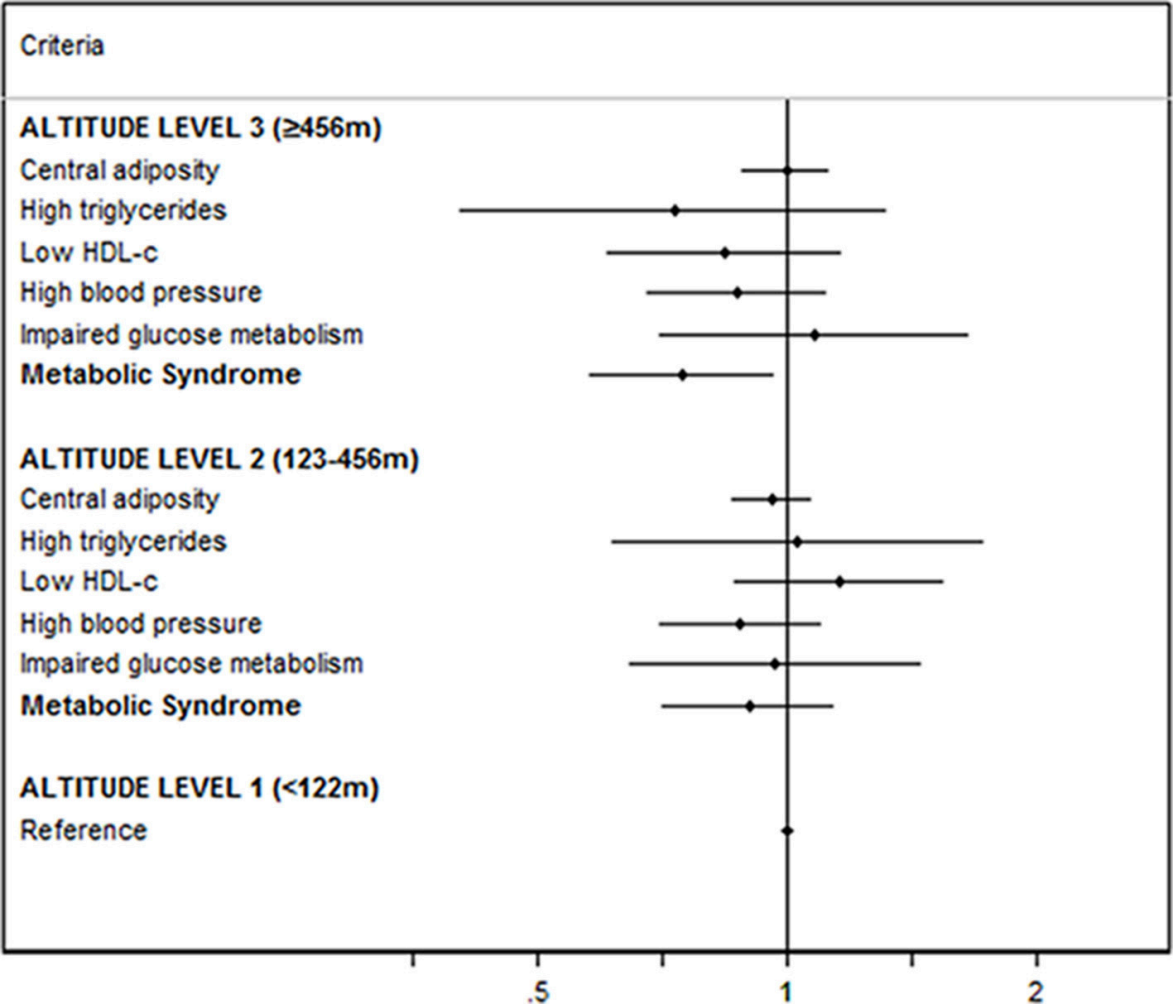
**Risk of developing each component of MetS according to the altitude level**. Multiple adjusted^*^ hazard ratios and 95% CI of incident MetS (harmonized IDF-AHA/NHLBI) according to the altitude of residence. The SUN Project 1999–2012. m, meters; ^*^ Adjusted for: gender, BMI, total energy intake, adherence to Mediterranean dietary pattern, physical activity, sedentary behavior, hours sitting, smoking status, snacking between meals, following special diets, sleeping time, and alcohol intake.

The risk of developing each component of MetS according to the altitude level is shown in Figure 2. The HR and 95% CI for the criteria of MetS were non-statistically significant after adjusting for potential confounders.

Four different definitions of MetS were used to study the hypothesis. These results, illustrated in Table 2B, were all in the same direction showing little variations among them. The reduction in the risk of developing MetS corresponded in all cases to the highest category of altitude compared to the first tertile, ranging from 23% lower odds in the IDF definition to 39% in the AHA/NHLBI definition as the greater effect.

**Table 2B:** **Comparison between MetS definitions according to different diagnosis criteria**.

	**Cases/ Person-year**	**Altitude levels**			***p* for trend**
		**0–121 m**	**122–456 m**	**457–2297 m**	
Harmonized	462/54,675	1.00 Ref.	0.90 (0.71–1.15)	0.75 (0.58–0.97)	0.029
ATP III	259/54,897	1.00 Ref.	0.83 (0.60–1.16)	0.66 (0.46–0.95)	0.024
IDF	441/54,702	1.00 Ref.	0.92 (0.72–1.18)	0.77 (0.59–0.99)	0.047
AHA/NHLBI	182/55,014	1.00 Ref.	0.82 (0.57–1.17)	0.61 (0.40–0.92)	0.017

Multiple adjusted^*^ hazard ratios and 95% CI of incident MetS according to the altitude of residence. Definitions abbreviations, harmonized of International Diabetes Federation and American Heart Association/National Heart, Lung, and Blood Institute (IDF-AHA/NHLBI); Adult Treatment Panel III (ATP III); International Diabetes Federation (IDF); American Heart Association/National Heart, Lung, and Blood Institute (AHA/NHLBI); m, meters; ^*^ Adjusted for: gender, BMI, total energy intake, adherence to Mediterranean dietary pattern, physical activity, sedentary behavior, hours sitting, smoking status, snacking between meals, following special diets, sleeping time and alcohol intake.

**Figure 3:**
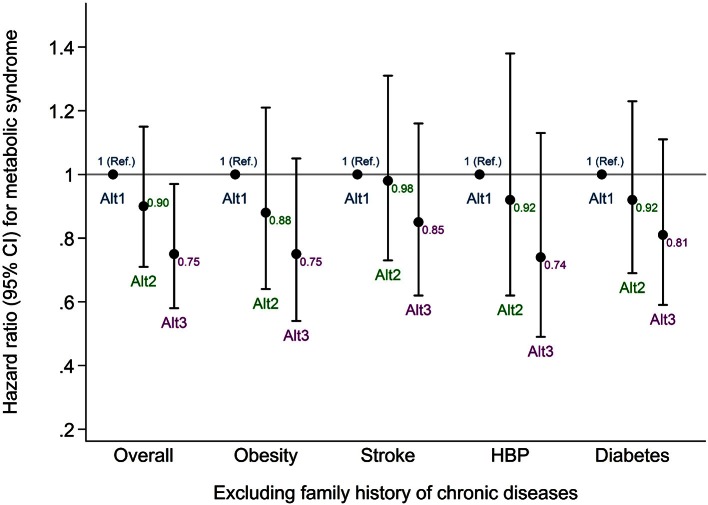
**Sensitivity analyses excluding subjects with family history of chronic disease**. Multiple adjusted^*^ hazard ratios and 95% CI of incident MetS (harmonized IDF-AHA/NHLBI) according to the altitude of residence. The SUN Project 1999–2012. Overall, all the participants included in the main analysis; HBP, high blood pressure; Ref, reference; Alt1, altitude tertile 1; Alt2, altitude tertile 2; Alt3, altitude tertile 3; ^*^ Adjusted for: gender, BMI, total energy intake, adherence to Mediterranean dietary pattern, physical activity, sedentary behavior, hours sitting, smoking status, snacking between meals, following special diets, sleeping time, and alcohol intake.

Furthermore, sensitivity analyses were in the same direction as the main results (Figure 3). The risk of developing MetS decreased in the higher altitude level after excluding participants with family background of chronic disease.

## Discussion

The current study showed that living at geographically higher altitude was significantly associated with a decreased risk of developing MetS in a Spanish population of relatively young university graduates. Moreover this effect followed a linear trend. The Cox model included age as the underlying time scale for all analyses. All models were stratified by years living in the city/village, categories of participants according to their date of entry into the cohort and categories of age (deciles). A model adjusted for gender, and two multivariable models were calculated. The first model with common dietary and lifestyle co-variables and the second model additionally adjusted for other potential confounders.

The higher concentration of oxygen (O_2_) that can be found in the earth corresponds to sea level (21%). At this altitude level the O_2_ partial pressure (pO_2_) is around 160 mmHg. Every 300 m reduces the pO_2_ 1% (McElroy et al., 2000; West, 2002). Our study suggests a potential preventive effect of living at moderately geographical altitude where the participants are exposed to lower pO_2_, pressure, humidity and temperature. In this sense, the prevalence of obesity has been positively associated with ambient temperature in two different observational studies (Valdes et al., 2014; Yang et al., 2015). It is also possible that other factors such as polymorphisms for high altitude adaptation were involved in this effect (Bigham et al., 2013; Huerta-Sanchez et al., 2013; Valverde et al., 2015). In our study the participants in the higher altitude category were at a median of 591 m and were exposed, for a median time of 20 years, to a standard barometric pressure of 95 kPa, which is 94% of the oxygen available at the sea level. This oxygen pressure translated to the body's oxygen supply taking 14 breaths per minute as the respiratory frequency and a tidal volume of 0.65 liters means an arterial pO_2_ of 12 kPa, and an oxygen saturation in blood (SaO_2_) of 97%. By contrast, the median altitude of those who were living in the lowest altitude (43 m) had an atmospheric pO_2_ of 100 kPa (with almost 100% of oxygen available at sea level). Using the same previous conditions this atmospheric pO_2_ means an arterial pO_2_ of 13.2 kPa and a SaO_2_ of 98% (Cruickshank and Hirschauer, 2004).

This difference of 1.2 kPa is not large, and these calculations have limitations due to the possible adaptive response over years and generations, nonetheless arterial hypoxemia induced by exercise is defined as a reduction in the arterial oxygen pressure only higher than 1 kPa (Nielsen, 2003). This leads to various physiological mechanisms following adaptation due to chronic lower hypoxia. In addition, from a population perspective we have to take into account the potential existence of Rose prevention paradox where factors that make a small difference to the population distribution may be more important than factors that have a clinical impact on a small number of people (Rose et al., 2008).

A significant reduction in the risk of developing MetS associated with living at geographically higher altitudes was found. However, this outcome was only statistically significant for the MetS as a whole but not for each one of its components separately. This lack of significance might be explained by the low incidence of the criteria of MetS in this young cohort (1.3% hypertriglyceridemia, 4.0% low HDL-c, 6.7% hypertension and 2.5% type 2 diabetes). Only central adiposity showed a relatively high incidence in our cohort (27.2%).

The reduction in the risk of developing MetS living at geographical high altitude followed a similar trend to the main analysis in those subjects with no family history of chronic diseases. These results suggest that the genetic background of chronic disease does not interfere with the potential benefits of living at geographical high altitude.

Our results are novel, but they are in line with other studies that did not specifically assessed MetS. Regarding to living at high altitudes, several observational studies have reported lower incidence rates of metabolic diseases in different large cohorts (Ezzati et al., 2012; Voss et al., 2013, 2014; Woolcott et al., 2014; Faeh et al., 2016). Obesity was significantly higher at lower altitude category (<500 m) in an US cross-sectional study after controlling for urbanization, temperature, behavioral and demographic factors (males and females had 5.1 and 3.9 times the odds of obesity, respectively, compared to participants over 3000 m) (Voss et al., 2013). A study about the incidence of overweight/obesity at geographical altitude performed in the SUN cohort found a reduction in the risk of developing overweight or obesity (Diaz-Gutierrez et al., 2016). Lately, a quasi-experimental study carried out with military population observed that individuals with frequent migration stationed at high altitude had 41% lower HR of obesity after multiple adjustment (Voss et al., 2014). Another trial, also performed in a US population, observed a lower risk of developing both obesity and type 2 diabetes (OR: 0.77 and 0.88 respectively) at high altitudes (from 1500 to 3000 m) (Woolcott et al., 2014). In addition, two studies found an inverse association between altitude (more than 1500 m) and mortality from ischemic heart disease, while a positive association was established between altitude and mortality from chronic obstructive pulmonary disease (Ezzati et al., 2012). The mortality rate from coronary heart disease was also reduced in men living above 1220 m (Faeh et al., 2016). These outcomes are consistent with our results, although they enrolled participants living at considerably higher altitudes than those in our cohort (up to 3500 m vs. <2000 m in our study). Higher hypertension prevalence was associated to highest altitudes (more than 2000 m) in highlanders of India (Norboo et al., 2015), which is opposed to our results concerning hypertension, possibly because their study was carried out with very high altitudes. As previously discussed, to date there are no longitudinal studies analyzing the association between living at geographical high altitude and the incidence of MetS.

Notwithstanding that metabolic syndrome definition is controversial (Balarini and Braga, 2016), we have considered the most widely accepted definitions of this concept to test our hypothesis. Comparisons conducted with different definitions of MetS showed similar results to the main analysis. However, diagnosis criteria influenced the association of altitude living and the risk of developing MetS, being AHA/NHLBI definition the most modified. This finding could be explained due to the central adiposity criterion, included in the AHA/NHLBI criteria, as high waist circumference (≥102 cm in males and ≥88 cm in females) or diagnosed type 2 diabetes (under drug treatment). AHA/NHLBI and ATP III share the cut-off point of waist circumference, as they do with the reduction in the risk of developing MetS (AHA/NHLBI 39% and ATP III 34% less odds). On the contrary, the other definitions (IDF and harmonized IDF-AHA/NHLBI) share the limit waist circumference (waist circumference: ≥94 cm in males and ≥80 cm in females) and their results were similar (Harmonized 25% and IDF 23% less odds).

Some limitations should be noted. Participants' high educational level increases the internal validity of the results, even though it could make our study population less representative. This feature might influence on their willing to participate in epidemiologic cohorts as they usually are healthier than the general population (Alonso et al., 2006). We should be cautious to generalize the results to the general population. A different sample may produce different results and therefore, it could be interesting to test our hypothesis in a different sample with participants other than graduates. However, several potential confounding factors related to lifestyle were used for adjustments. Another potential limitation is the way of collecting data relative to altitude, as it was only measured at baseline assuming that the participants remained at the same altitude over the follow-up period. Moving to a different altitude might be a limitation in our study, since some participants may have changed their permanent residence. Moreover, the exposure variable has a limiting factor in this study since in Spain there are few people living over 1500 meters. Atmospheric characteristics (temperature, humidity or pressure) and population density-related features (pollution from urban areas, migrations or food accessibility) were not available in our study, but they could be involved in the effects of living at geographical high altitudes. These data would help to clarify the role of environmental factors in the development of chronic diseases; nonetheless urban areas are more or less well distributed due to the presence of big cities both in the lower and in the other two tertiles of altitude. Finally, some clinical parameters could have helped to avoid potential confounders as self-reporting could lead to bias. Nevertheless, participants were not aware of the objective of the study when they answered the questionnaires of the project. Finally, although we have adjusted for potential confounders, we cannot rule out the existence of residual confounding.

Major advantages are its prospective design with at least 6 years of follow-up to avoid reverse causation bias, the previously validated methods to assess the main variables (Barrio-Lopez et al., 2011), the use of a large sample of participants and their university education, which helped them to provide a high quality of information in their answers to the questionnaires and also adds homogeneity to the cohort regarding socio-demographic factors. These features of the SUN cohort may reduce the potential for confounding.

## Conclusions

In this study of a Spanish population composed of university graduates, we found that to live at geographically higher altitude was directly associated with a lower risk of developing MetS after 6 and 8 years of follow-up. This association was only significant when MetS was analyzed as a whole. However, this is a preliminary result and future studies with longer follow-up periods, larger sample size and higher altitudes are needed. Additional research, even including the future (but remote) possibility of conducting a randomized preventive intervention, should clarify the mechanisms of this relationship, and assess the pros and cons of geographical high altitude residency on MetS prevention. Moreover, our results need further validation in the general population. Up to date, this is apparently the first large longitudinal study analyzing the association between MetS and altitude. Our findings suggest that geographical elevation may be an important factor linked to metabolic diseases.

## Author contributions

Conceived and designed the study: AL, PG, AM. Managed the data set including data preparation, performed data analyses and assisted with interpretation of the study findings: AL, MB, CS, AP, JD, JP, MM, PG, AM. Analyzed the data: AL, AP, MB, MM, PG, AM. Contributed to writing and revising the manuscript: AL, MB, CS, AP, JD, JP, MM, PG, AM. All authors read and approved the final manuscript.

## Funding

The SUN Project has received funding from the Spanish Government Carlos III Health Institute Centre of Biomedical Research Network: CIBERobn Physiopathology of Obesity and Nutrition, and the European Regional Development Fund (FEDER) (RD 06/0045, CIBER-OBN, Grants PI10/02658, PI10/02293, PI13/00615, PI14/01668, PI14/01798, PI14/01764, and G03/140), the Navarra Regional Government (45/2011, 122/2014), and the University of Navarra.

### Conflict of interest statement

The authors declare that the research was conducted in the absence of any commercial or financial relationships that could be construed as a potential conflict of interest.
